# Senescence-inhibitory Δ133p53α counteracts accelerated ageing and mortality

**DOI:** 10.64898/2025.12.31.697195

**Published:** 2026-01-20

**Authors:** Leo Yamada, Huaitian Liu, Natalia von Muhlinen, Curtis C. Harris, Izumi Horikawa

## Abstract

Research on progeria not only contributes to treatments for the disease but also enhances our understanding of physiological ageing ^1^ . Mouse models of progeria recapitulate pathological ageing phenotypes seen in patients, including cardiovascular defects, increased cellular senescence, systemic inflammation, DNA damage accumulation, and shortened lifespan ^2^ . In cultured cells from Hutchinson-Gilford progeria syndrome (HGPS) patients, the human p53 isoform Δ133p53α was previously shown to inhibit p53-mediated cellular senescence, proinflammatory IL-6 production, and DNA damage accumulation, and to extend cellular replicative lifespan ^3^ . Here we show that, in a heterozygous HGPS mouse model ^4^ , transgenic expression of Δ133p53α reproduces these *in vitro* -observed effects across multiple organs *in vivo* and extends median lifespan by 11% (387 versus 349 days, *P* = 0.0379). In the aorta and skin, Δ133p53α abrogates progeria-characteristic pathological changes and preserves tissue integrity. Our data further suggest that Δ133p53α may promote a broad spectrum of ageing-counteracting mechanisms, including bone homeostasis, metabolic fitness, antioxidant defense, youthful epigenome, and tissue stemness. Together with the anti-inflammatory and tissue-preserving effects of Δ133p53α in naturally aged mice and its age-associated downregulation in human tissues, this study suggests that Δ133p53α-based therapeutic strategies may be applicable not only to HGPS but also as broader interventions for preventing or delaying ageing.

## Full Text Availability

The license terms selected by the author(s) for this preprint version do not permit archiving in PMC. The full text is available from the preprint server.

